# Healthcare Professional and Service User Perspectives on Formal Educational Programmes for Children and Young People with Cancer in the UK

**DOI:** 10.1007/s13187-020-01854-7

**Published:** 2020-10-06

**Authors:** Wendy McInally, Karen Campbell

**Affiliations:** 1 Faculty of Wellbeing, Education and Language Studies, The Open University, 10 Drumsheugh Gardens, EH3 7QJ Edinburgh, Scotland; 2 Macmillan Cancer Support, Caledonian Exchange, 19A Canning Street, Edinburgh, EH3 8EG Scotland

**Keywords:** Children, Young people, Healthcare professionals, Education and training, Mixed methodology

## Abstract

Caring for children and young people with cancer requires specific knowledge, skills and experience to deliver the complex care regimes both within the hospital or community environment. This study explored the educational gaps in caring for children and young people with cancer. To address this, a mixed methodology approach was adopted in two phases. Phase one was a questionnaire circulated to healthcare professional members (*n* = 850) of the Children’s Cancer and Leukaemia Group and Managed Service Network, Scotland. Response rate (*n* = 121) (14%) was achieved. In phase two of the study, a focus groups (*n* = 4) was conducted with young people in Scotland through the Managed Service Network. This was to gain a critical understanding from service user perspective and what they deemed as important to their overall care delivery. Phase one: healthcare professional results reported that 76% (*n* = 93) were aware of education; 69% (*n* = 84) found that knowledge supported practice development, but only 45% (*n* = 55) finding current education provision useful. The top education topics identified to be lacking in educational availability were communication, psychological support, dealing with young people, supportive care, diagnosis and treatment and challenges to learning. Several participants 64% (*n* = 78) suggested that funding and time was a barrier, and that there was a lack of provision. Phase two: Findings from the focus group (*n* = 4) thematic analysis identified five key themes. Service users expected professionals to be knowledgeable and trained, but when talking about experiencing care, gave insights into the gaps in their care. Findings suggest that formal cancer education is required.

## Introduction

Worldwide, cancer is a major concern; therefore, education and training of healthcare professionals is of the utmost importance moving forward. It is also essential to understand what is necessary within the care pathway for children, young people and their families. Therefore, involving service users where appropriate is vital in addressing these needs [1, 2].

Cancer in children and young people is rare compared with adult cancers but is an important cause of mortality and morbidity in all age groups. Every year in the United Kingdom (UK), approximately 1 in 600 children will develop cancer in the first 15 years of life [1, 2]. This equates to approximately 1400 new cases of cancer in children each year in the UK and 1900 15- to 24-year olds [3, 4]. Normally all new cases are diagnosed at the regional specialist centre, then depending on the treatment protocol and the needs of the child, young person and family, treatment will be delivered within the local district hospital or home environment [5].

Over the past 40 years, the 5-year survival rates for children and young people with cancer have risen dramatically in the UK [6]. This improvement is largely attributable to the treatment therapies along with the centralisation of care. Policy within the UK now enables all children and young people to receive agreed treatment protocols underpinned by national and international studies or controlled clinical trials [1, 4, 6] and is provided throughout the UK in regional children’s primary treatment centres, Teenage Cancer Trust (TCT) Units and or adult specialist centres. Treatment usually consists of the introduction of intensive multi-agent chemotherapy, combined with radiotherapy, surgery and immunotherapy as required [6].

Within the UK, children are normally managed in children’s services from the age of 0 to 14 years. Young people 15 to 24 years are nursed either in TCT units or in adult services. It is worth noting that there is no universal definition of a young person as the age parameters differ from within the literature around the world [7]. Cancer accounts for less than 1% of illnesses in children and young people; however, it is the leading cause of death for this age group in the developed world [3]. The main types of cancer in children are leukaemia, brain tumours and lymphomas, whereas in young people, the main types of cancer are carcinoma, lymphoma, and brain, spinal tumours and skin cancer [3, 4].

Children and young people’s cancer have unique qualities that have attracted healthcare professionals to work within this speciality over the years [8]. With the advances in treatment, increasing survival rates, there is a need for more expert specialist care. Current education and training provision for all levels of cancer care within the UK is varied and fragmented [9–11]. As the speciality grows, healthcare professionals have been keen to learn new knowledge and skills, but this has not translated to access to educational-accredited programmes.

### Research Aims and Objectives

The aim of this study was to establish what education and training is available across the UK and what specific education and training is required to care for children and young people with cancer from a patient perspective.

- Dual approach of conducting a national evaluation of education and needs of healthcare professionals and service users;
- From this establish recommendations for education and training as required.

## Method

### Data Collection

A mixed-methods approach was undertaken to explore the healthcare professional and young people’s perspectives of necessity for cancer education and training for children and young people’s provision of care. These methods were chosen as they offered convenience to explore this area with established groups within the UK [12]. Phase one consisted of an online questionnaire which was conducted with a convenient sample of healthcare professionals through CCLG and Managed Service Network (MSN). The MSN, for Children and Young People with cancer, is a national network of healthcare specialists from different National Health Service boards across Scotland. Phase one findings informed the questions for the focus group. Fifteen open and closed questions were developed from phase one and from the literature relating to cancer education for children and young people in the UK, based on the Kirkpatrick model (1996), [13]. Questions were formulated to determine characteristics of the population, speciality and level of education around what they perceived was missing. The structure and style of the questionnaire was based on previous work undertaken by the researchers [10] and using a web-based survey software tool hosted by the University to enable collation of data from participants. The survey opened on 5 February and closed on 13 June 2017, and potential participants received two reminders before the closing date.

Results from phase one revealed five themes: communication, psychological support, dealing with young people, supportive care, and diagnosis and treatment. These themes were then populated within the focus group semi-structured interview schedule. Phase two was a focus group with young people (*n* = 4) from a youth advisory forum group and the support of the Lead Nurse (LW). Although ten (*n* = 10) participants were invited, only four (*n* = 4) participated. It is important to highlight that young people under the age of 16 years of age were not included as it may have been a challenge for them to answer the questions posed to them [14]. In phase two, the focus group was undertaken in October 2017, with young people with a diagnosis of cancer. The intention of the focus group was to ascertain service user’s perspective of educational needs of healthcare professionals and to involve them within the research [2]. The initial response was that they had no perspective other than they should (healthcare professionals) have the knowledge and training. Therefore, the research interviewer (KC) changed the emphasis of the questions to asking about experience of care to elicit care deficits for analysis. Focus groups over the last 15 years have gained acceptance within healthcare research and are popular in qualitative studies as they are designed to obtain in-depth information in a non-threatening way [15]. The focus group was arranged at a date and time that was convenient to the young people. It lasted 60 min and was digitally recorded. It was facilitated by two researchers (KC and W McI), one of whom took notes (WMcI).

### Sample

**Fig. 1:**
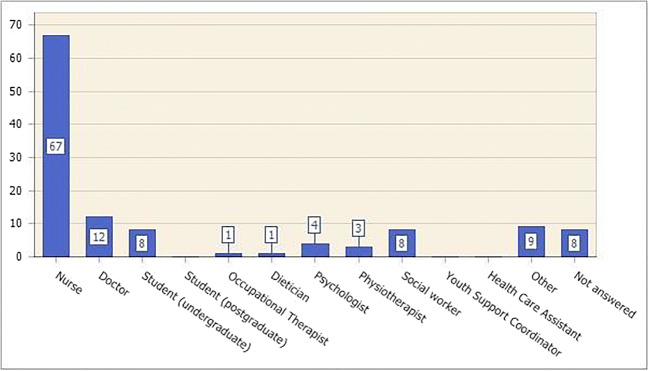
Findings from the quantitative data

A convenience sample of healthcare professionals across the UK was invited to participate who were part of the CCLG/Royal College Nursing (/RCN) and MSN. These organisations comprised of a total of 850 members, (*n* = 773 from the CCLG, *n* = 77 from MSN). The members were approached through email by an independent administrator alerting them to the survey. Of the 850 potential participants, 121 (*n* = 121) completed the questionnaire giving a response rate of 14.2%. Findings from the quantitative data are reported in Fig. 1 which identifies the healthcare professionals that participated in the questionnaire. As can be seen, nurses were the most significant healthcare professionals that completed the questionnaire. The sample consisted of 67 nurses (*n* = 67), 12 doctors (*n* = 12), eight trainee medical students (*n* = 8), eight social workers (*n* = 8) and an array of allied health professionals. The participants for the focus were young people who were part of the MSN. Although focus groups usually comprise 8 to 12 people and this was originally the intention, only *n* = 4 were able to participate in the work [12, 15, 16]. Nevertheless, the literature suggests that smaller focus groups enable rich data to be extracted and adds to the rich tapestry of knowledge [12]. The environment was calm and quiet and allowed the participants the privacy required.

### Data Analysis

Quantitative and qualitative data were collated through the questionnaires, whilst only qualitative was collected in the focus group. Data analysis was undertaken by all researchers, one of whom was independent of the study (DW). The quantitative statistics were analysed using descriptive statistics through the online Novi survey tool. The qualitative data were transcribed verbatim using thematic analysis [15]. The transcripts were read until both researchers were familiar with the content, after which codes and themes were drawn up for discussion. The themes were compared, and any disagreements were discussed in more detail; where resolution could not be resolved, these and the rest of the themes were discussed with the other researchers. Data analysis was performed separately on the questionnaire and the focus group. The team (W McI, KC and DW) then conducted a cross-analysis and mapped the themes to derive overlapping concepts which will be presented in this paper.

### Ethical

University ethical approval was granted from the School of Health and Social Care ethics at Edinburgh Napier University committee in the January 2017. All participants received an information leaflet explaining the purpose of the work and that all responses would be confidential. Participation in the study was voluntary, and appropriate consent obtained from individual participants throughout the Novi tool. All participant involvement was anonymous (RCN). Only the researchers had access to the questionnaire data which was password-protected.

For the focus group, the study was explained to the participants on the day to ensure they understood the purpose of the work. They were given opportunities to answer questions, before written consent was taken. All participants were over 16 years of age and therefore could give their own consent. It was made clear to the participants that they could withdraw at any time, and that their rights would not be challenged. However, if they wanted to withdraw during the focus group, the tape could not be destroyed as there were other voices contained within this, but assurance was given that their contribution would not be used.

## Survey Findings

There was an array of cancer education available for practitioners across the UK. The highest percentage of education was in-house study days (37%) followed by postgraduate education (18.4%). The other results were made up of short online course accredited and non-accredited courses (17.4%), undergraduate courses (8.9%) and other (1.6%). Of those who responded, 76.9% said that they were aware of cancer education resources compared with the 14% who were not aware of any resources. In relation to questions relating to the value of education, 67.2% felt education was valuable, whereas the impact on practice tended to centre on communication, confidence and knowledge.

**Fig. 2:**
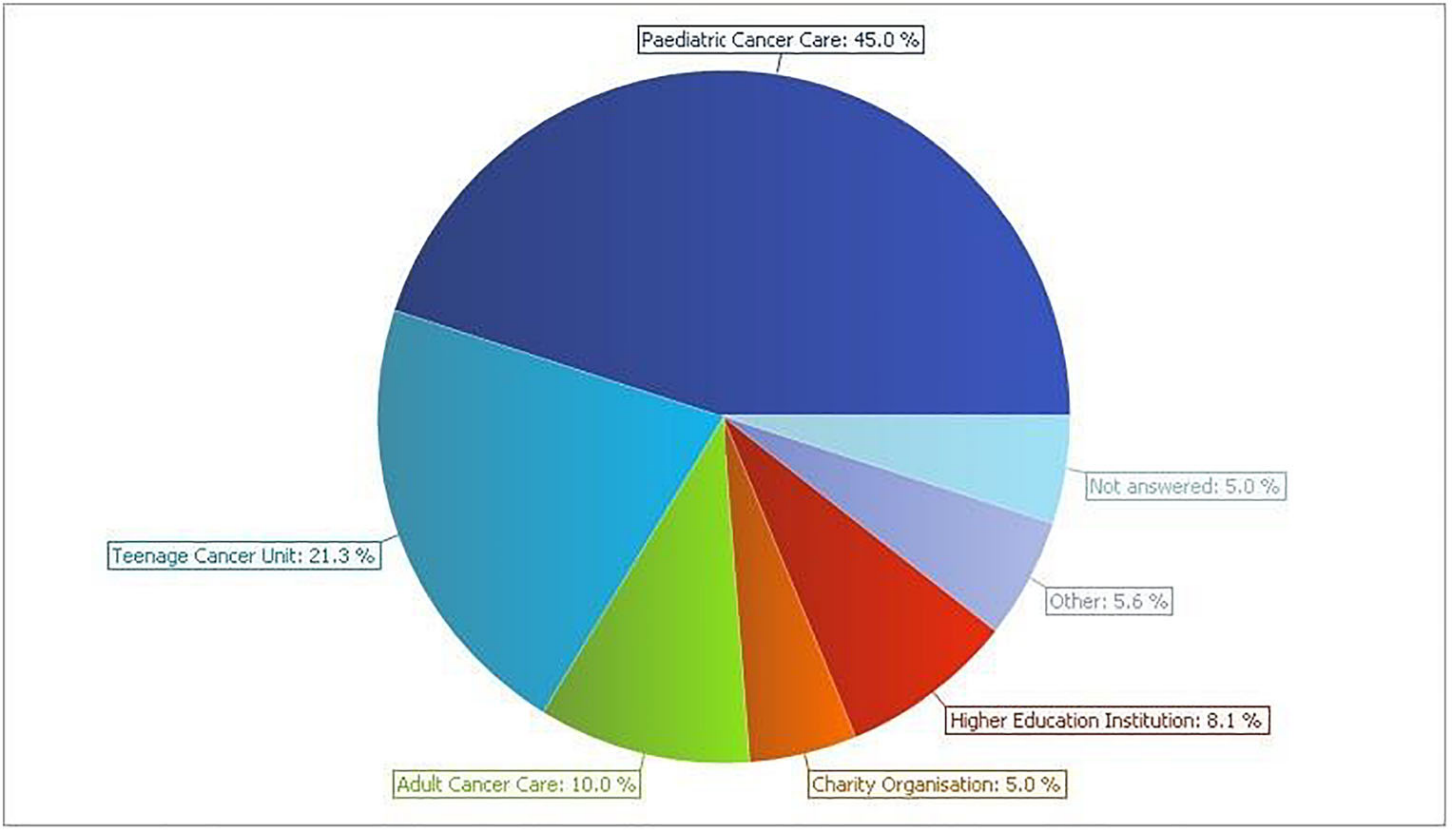
The areas where the participants worked

The areas the participants worked can be found in Fig. 2. Most participants worked within the area of paediatric cancer care, followed by the Teenage Cancer Unit.

Through asking questions such as “what education in the care of children and young people with cancer would support you within this speciality”, it became apparent that the participants wanted more accessible education. There were key accredited courses, and although some were regarded as online credited modules, there was still a request to have this more local to the participant’s country and possibly healthcare system or provision for young people’s services. In particular, knowledge and skills in leadership and mentorship within a multidisciplinary setting would be advantageous. From a topic perspective, it was apparent that treatment-related acute and late effects and chemotherapy and bone marrow transplant were the most cited. Supportive and palliative care were also topics of interest mainly in psychological aspects of care and nutrition. One participant mentioned skills based around coaching for health behaviour, and unpicking fact from fiction and communication in general was cited by two participants. A further two participants mentioned age-appropriate communication to support teenagers.

Taking the issue of access further, the participants highlighted funding, study leave, time pressures, staff shortages and work-life balance. When questioned on attending the courses/sessions, the overwhelming result was positive with some key supportive considerations from practice. A number of participants acknowledged that there were challenges in implementing education, if the service was new or adult-orientated. Challenges to implementation were recognised through supportive leadership, management and robust governance processes. Participants acknowledge that “the doing” of assessment encouraged implementation. From the thematic analysis, of the qualitative narrative from the questionnaire, five key themes emerged: supportive care, TYA, communication, diagnosis and treatment and challenges to learning. These themes supported the structure of the interview schedule for the focus group with the young people.

### Focus Group Findings

The focus group consisted of two (*n* = 2) females and two (*n* = 2) males aged between 16 and 24 years of age. They all came from different regions of Scotland and had been or were being treated for cancer within different primary care centres or shared care. There were five (*n* = 5) themes which emerged, and although they were similar to the key findings from the qualitative part of the questionnaire, there were distinct differences from what the young people expect from the care received from healthcare professionals and what they thought was important from their own experiences.

### Theme 1: Nurse Education

Education and training was suggested as essential but something that the young people had not given much thought to as they just expected that this would be a given. This theme emerged from a sample of four participants (*n* = 4). They all had different personal experiences within either a specialist or a non-specialist centre, but all had experiences of care from all healthcare professionals, such as the doctor, nurse, psychologist and dietician. As one participant described:

“I liked it when you are in hospital because you’re getting looked after by a number of people, and you think that they know everything, so you feel safe and things aren’t going to go wrong” (P1).

The participants described an overwhelming response to one of the first questions by the researcher (KC).

“Did you all have an expectation that healthcare professionals are educated in what you have and how to care for you?”; (KC) by responding “You just trust them and that they have the knowledge” (P3) and “haven’t got a clue, but I guess they go to training” (P4).

### Theme 2: Communication

Participants reflected on their own experiences of their cancer journey and how important parts of this were communicated to them as one young person reported, “So, I don’t know if they are taught on how to communicate’ and there is definitely a place for paediatric oncology education but there was very little clear information given for teenagers especially at time of diagnosis. It was evident that communication is vitally important but not always a positive aspect of the cancer journey” (P1).

Another recalls, “I’ve had doctors that have been better or more personal than others which is important for my parents” (P2). This theme highlights the need for effective communication especially at the time of diagnosis and ongoing physical, emotional and social support.

### Theme 3: Specialist and Non-specialist Practice

This theme emerged which clearly revealed that the young people all had different experiences and were cared for within different clinical environments.

“Without the TCT I don’t think that there would be as much follow up care, kind of advice. I mean, some of us have been on treated for our cancer but ‘what to do now” (P1).

The delivery of high-quality evidence-based care by healthcare professionals who are knowledgeable and experienced within the speciality of cancer was a fundamental requirement to the cancer experience. Given a choice was also key to their journey. As one participant described:

“I was given the choice of being treated in an adult ward at a different hospital closer to home or at the specialist unit. But because I was diagnosed in the specialist unit, I’d seen what was available and I’d meet the team that I’d be with, so I decided to just travel a bit further “(P3).

There is a tendency to view the ideal treatment and care experience as arising out of specialist services that are age-appropriate. This however is not readily available for all young people with cancer across the globe due to different healthcare systems and resources, but it was clear from this focus group that the young people were prepared to travel.

### Theme 4: Treatment and Being a Young Person

The impact of cancer on the young person was emphasised throughout, and it became clear that young people living with cancer experience uncertainty about their future. As one other participant explained “My mum and dad stop working so we had the time but it was really like your whole life was put on hold” (P1) and “at that age where we still want our family there but we also want our independence being able to sign our own consent forms and things, and I think that doctors and nurses maybe have to realise that too. Everyday I’m waking up thinking of cancer, going to bed thinking of cancer, wanting to do well in class because of cancer” (P2).

Cancer brought many challenges which were out of their control. The impact of cancer on the young person and their family brought feelings of uncertainty about the future, hope for recovery and life itself.

### Theme 5: What is Missing

Young people were able to articulate what they felt was missing from the care delivery they had received. As one participant expressed “I want to try and change nutrition in hospitals. So, it’s kind of an on-going thing, it’s not really back to normality” (P3). He felt that nutrition was important in his full recovery and that this was an aspect of care often left too late to be taken into consideration.

It was also clear that young people wanted health and social care professionals to effectively communicate their cancer diagnosis and treatment with them rather than their families. The delivery of high-quality evidence-based care, by health and social care professionals who are knowledgeable and experienced within the cancer speciality and the immediate and long-term implications a cancer diagnosis brings, was a fundamental requirement to the cancer journey.

Table 1 highlights the main areas of cancer education that both the healthcare professional and young person suggested was missing.

**Table 1:** The main areas of cancer education

Questionnaire	Focus group interviews themes
Plethora of course available in the form of university online and face-to-face accredited courses, e-learning, conferences, study days and webinars. Network learning groups. Mentoring. However, distribution across the country is unequal.	Nurse education was not a concept to be thought about just that they should be trained. Limited university education provision in area of focus group.
Communication was highlighted in a few responses as an element of education required. Chemotherapy administration, disease-specific supportive palliative care. Heath-related behaviours	Communication: this theme showed more prominence as an issue with the service users over other elements of safety which was a given.
No differentiation between specialist versus non-specialist education. However, this is only 13.2% of participants worked in the adult care sector, with 87% (*n* = 106) working in a cancer age–specific sector.	Specialist versus non-specialist
Similarly, the developmental nature was identified as an education requirement from healthcare professionals.	Treatment and being a young person
Healthcare professionals concentrated on access issues of education and lack of provision across the UK. Again, more speciality-driven rather care within adult services. More about clinical decision-making and late effects. Multidisciplinary sharing of case studies.	Missing topics of education Nutrition Family-centred care

## Discussion and Conclusions

It is clear from this study that healthcare professionals working within this specialist area of care welcome resources to enable them to provide the best possible care for children and young people with cancer and their families. However, although not a comparative study, it was evident that education and training varies and is inconsistent across the UK [9–11, 17]. For those working in these areas, they appreciated that they needed to be prepared both educationally and practically hence required the knowledge and skill set to care efficiently and effectively for this patient group and their families. Healthcare professional’s welcome education and training that included or excluded accreditation but had financial implications. There were however clear areas of learning within children and young people’s care that require to be developed for healthcare professionals across the UK.

New approaches to the ways in which learning, teaching and assessing are considered are being used to ensure life-long learning. Children and young people with cancer are a unique speciality and one that has seen many changes over the past 40 years [18–20]. The education of healthcare professionals specialising within these areas has become paramount in these constantly evolving specialities [19]. Working within specialist or non-specialist settings where children and young people with cancer are being cared for, the environments are facing fresh challenges ahead, especially as the survival rates continue to improve [1, 2]. As these improvements develop, ongoing education and training is essential and necessary to assist in the effective delivery of treatment and supportive care regimes [1, 4].

Although the topics for education seemed divergent from the focus group data, there were key similarities. The divergence only came from operationalising of care and the system within which they delivered that care. This transpired as treatment administration and knowledge about acute and late effects of care; how to implement innovation with managerial support; and correct governance structures. The focus group expressed this knowledge to provide safe and effective care; in other words, this was a given when being treated for cancer. Similarities were apparent in identification of communication and delivering care in the context of knowing “the teenager” as well as treating the cancer. In the focus group, this was for both those that were treated in a specialised cancer unit or on an adult ward.

The experience of cancer can often occur at a time when young people are in the process of developing their early adult life plans, transitioning from being a child to teenager and from teenager to young adult [20]. Researchers need to be aware that participants may not see the world from their perspective, and therefore, future researchers need to take this on board. Involving children and young people within research allows them to have a voice and be instrumental in their care delivery [2, 20]. Family-centred care (FCC) has for many years been the pillar of children and young people’s nursing in the UK and some parts of Europe [21–23], and person-centred care (PCC) within adult nursing practice [24]. This study would suggest that both FCC and PCC are important concepts and present themselves within different parts of the cancer journey.
